# Unravelling the antitumor mechanism of Ocoxin through cancer cell genomics

**DOI:** 10.3389/fphar.2025.1540217

**Published:** 2025-03-19

**Authors:** Iera Hernandez-Unzueta, Uxue Telleria-Gonzalez, Ana María Aransay, José Ezequiel Martin Rodriguez, Eduardo Sanz, Joana Márquez

**Affiliations:** ^1^ Cell Biology and Histology Department, Faculty of Medicine and Nursing, University of the Basque Country, Leioa, Spain; ^2^ Genome Analysis Platform, CIC Biogune, Derio, Spain; ^3^ CIBERehd, Instituto de Salud Carlos III, Madrid, Spain; ^4^ Catalysis S.L., Madrid, Spain

**Keywords:** Ocoxin, cancer, plant extract, ferroptosis, adjuvant, chemotherapy

## Abstract

Cancer is one of the leading causes of death worldwide. Many therapies are being used to treat this disease, however, new treatments are now being implemented, since they are not always effective and their secondary effects represent one of the main reasons for cancer patients’ loss of life quality during the progression of the disease. In this scenario, Ocoxin is a mixture of plant extracts, amino acids, vitamins and minerals, known for its antioxidant, anti-inflammatory and immunoregulatory properties, which has shown to exert antitumor effects in many cancers. The aim of this study is to elucidate the mechanism of action of the compound in colorectal cancer, triple negative breast cancer, pancreatic cancer and prostate cancer. Analyses performed through RNA sequencing revealed that the main effect of Ocoxin appears to be the alteration of cell metabolism, especially inducing the process of ferroptosis. Nevertheless, the modulation of the cell cycle was also remarkable. Ocoxin altered 13 genes in common in all the four cancers that were not only associated to metabolism and cell cycle but were also involved in the integrated stress response and unfolded protein response, suggesting that the compound causes the induction of cell death through several pathways. Although the mechanisms vary according to the type of cancer, this study highlights the potential of Ocoxin as an adjunctive treatment to improve outcomes in cancer therapy.

## 1 Introduction

Cancer poses a major challenge in our times, as it affects individuals all over the world, regardless of age, sex, race or socioeconomic status. Advances in research have led to a reduction of cancer-related deaths thanks to early detection methods and the improvement of treatments. However, cancer still poses a challenge to current medicine due to the limitations of therapies like toxicity and the fact that they occasionally become ineffective since tumors can develop resistance to them over time. In this context, radiotherapy and chemotherapy are the most extended remedies, which help to control tumor growth by destroying cancer cells, although they cause toxicity and weakness, resulting in a deterioration of patient’s health status. Additionally, less invasive methods are available, such as immunotherapy and targeted therapy, which reinforce and restore the immune system and target specific molecules, respectively. Still, these treatments can also cause side effects and tumors can develop resistance to them (Sarmento-Ribeiro et al., 2019; Labrie et al., 2022). Thus, further research is needed to develop more efficacious and less harmful treatments. As an alternative to prevent resistance and toxicity, combinations of several compounds are being administered to patients, which work together against cancer, each bringing their own unique strengths to bear on the disease (Woodring et al., 2023; Vladu et al., 2022). In this context, plant extracts obtained from seeds, leaves, roots or fruits are currently under investigation due to their demonstrated antitumor properties that target both cancer cells and the tumor microenvironment (Choudhari et al., 2020; Hosseini and Ghorbani, 2015; Pan et al., 2019; Rana et al., 2021; Xu et al., 2016; Khan et al., 2019; Masuelli et al., 2021). In fact, countless works report the capacity of flavonoids, polyphenols, vitamins, minerals and many other bioactive compounds to reduce tumor growth through numerous mechanisms, such as promoting apoptosis, blocking cell cycle checkpoints, inhibiting cell proliferation, reducing the migratory capacity, modulating the immune response and reducing the secretion of pro-tumoral factors, among others (Esmeeta et al., 2022; Lopes et al., 2017; Reed et al., 2018; Kim et al., 2021; Masuelli et al., 2021; Almatroodi et al., 2020; Khan et al., 2019; Zhang, W. et al., 2022; Zhang, Y. et al., 2023; Wang W. et al., 2023). In this regard, the possibility of combining first-line therapies used in clinics with these products is being studied. Indeed, certain natural compounds and their mixtures are already being administered to patients’ as adjuvants together with antitumor treatments, as they can enhance their effect (Harvie, 2014; Lee et al., 2021; Wang, S. et al., 2024; Farghadani and Naidu, 2022; Shi et al., 2023; Ng et al., 2022; Abadi et al., 2022; Talib et al., 2022; Chen, Z. et al., 2023). Besides, these supplements, as well as supporting nutrition, can help to mitigate adverse effects of treatments, such as loss of appetite, nausea and fatigue, thus improving tolerance to therapy and the patient’s quality of life (Harvie, 2014; Liu et al., 2021; Wang, K. et al., 2022). Green tea, for instance, is one of the most widely consumed beverages in the world that possesses many health benefits and may serve as a preventive element for cancer development. Its polyphenols, especially the epigallocatechin-3-gallate (EGCG), are known to protect against inflammation, inhibit tumor cell growth, modulate the secretion of metalloproteases and reduce the expression of immune checkpoint inhibitors, among others (Masuelli et al., 2021; Almatroodi et al., 2020). Similarly, many spices have shown antitumor effects against cancer: cinnamon induces apoptosis, impedes cell growth and hinders angiogenesis (Peng et al., 2024; Sadeghi et al., 2019); curcumin suppresses proliferation, enhances apoptosis and triggers mitochondrial stress in tumor cells (Abadi et al., 2022); ginger reduces cell viability, boosts cell death and modulates cytokine secretion (Mahomoodally et al., 2021); licorice also induces apoptosis, inhibits angiogenesis and impedes cell migration and invasion (Zhang, Y. et al., 2023); turmeric inhibits cell proliferation, promotes apoptosis and cell cycle arrest, and blocks Epithelial-Mesenchymal Transition (EMT) and invasion (Zhang, Y. et al., 2023; Wang X. et al., 2023). Likewise, some fruits like berries, grapes and many others, have shown beneficial effects against cancer by increasing cell death, causing cell cycle arrest and by reducing inflammation and the presence of Reactive Oxygen Species (ROS) (Masuelli et al., 2021; Esmeeta et al., 2022).

Related to this, Ocoxin is a mixture containing a combination of plant extracts (green tea, licorice, cinnamon), amino acids, minerals and vitamins. Although the compound was originally designed as a supplement to strengthen the immune system and to improve the general wellbeing of patients suffering from cancer and promote their overall health, several studies have reported its antitumor mechanisms. Briefly, Ocoxin showed to reduce tumor growth *in vitro* by decreasing cell proliferation, blocking cell cycle, augmenting apoptosis, modulating gene expression and altering the secretome of tumor cells while reducing chemoresistance and improving the effect of different chemotherapeutic drugs. Moreover, *in vivo* studies performed in mice revealed that, apart from reducing tumor volume, the natural supplement modulated the expression of secreted cytokines, diminished the infiltration of macrophages and Cancer Associated Fibroblasts (CAFs) and reduced angiogenesis (Hernandez-Unzueta et al., 2023; Hernandez-Unzueta et al., 2017; Benedicto et al., 2021; Márquez et al., 2016; Hernandez-Unzueta et al., 2019). Furthermore, several clinical trials have indicated the advantages of using Ocoxin together with cancer standard care therapies to alleviate side-effects and to improve the quality of life of patients (Shumsky et al., 2019; Fundora-Ramos et al., 2021; Kaidarova et al., 2019).

As we mentioned, extensive research has been performed on the antitumor effect of Ocoxin against both tumor cells and tumor microenvironment, which has permitted the comprehension of the biological processes in which the compound participates. However, much remains to be discovered about the underlying mechanisms and altered pathways that may be involved in the response to the treatment with Ocoxin. In order to gain a more complete understanding of how the natural mixture acts in the context of cancer, we have chosen to carry out RNA sequencing of the four cancer types mentioned above. This approach will allow us to identify which specific genes are altered in response to the compound and, in turn, help us to infer which biological pathways might be modified. This more detailed analysis will permit us to gain crucial insights into the possible mechanisms of action of Ocoxin in cancer and may provide a solid basis for future clinical studies and therapeutic developments.

## 2 Materials and methods

### 2.1 Cell culture

Four different human cancer cell lines were cultured to carry out this study: the colorectal adenocarcinoma (COAD) SW620 cell line, the pancreatic adenocarcinoma (PAAD) BxPC-3 cell line, the prostatic adenocarcinoma (PRAD) LNCaP cell line and the triple-negative breast cancer (TNBC) HCC-70 cell line (ATCC, Manasas, VA, United States). All the cell lines were cultured with RPMI-1640 medium supplemented with 10% Fetal Bovine Serum (FBS) and Penicillin-Streptomycin-Amphotericin B solution (Gibco, Waltham, MA United States) at 37°C and 5% CO2 in a humidified atmosphere.

### 2.2 Viability assays

In order to assess the effect of Ocoxin against the mentioned cancer cell lines, several viability assays were carried out. To start with, 7 × 10^4^ cells/mL were cultured onto 96-well plates under standard conditions. After 18 h, culture medium was removed and cells were treated with Ocoxin diluted (1:1000; 1:700; 1:500, 1:200, 1:100, 1:50, 1:25) in RPMI-1640 supplemented with 1% FBS for 24 h. Then, fresh Ocoxin was added again to the cells for another 24 h and cell viability was measured in the Fluoroscan Ascent (Thermo Labsystems, Waltham, MA, United States) with the Cell Counting Kit-8 (Sigma-Aldrich, St. Louis and Burlington, MO, United States) according to the manufacturer’s instructions. Finally, the half-maximal inhibitory concentration (IC50) of Ocoxin was established in all the cell lines to perform the following experiments.

### 2.3 mRNA sequencing for cancer cell gene expression analysis

Genetic alterations induced by Ocoxin were analyzed through mRNA sequencing in the previously mentioned human cancer cells. First, cells were cultured in 6-well plates under standard conditions at a concentration of 1.5 × 10^5^ cells/mL. After 18 h, each cell line was treated with the IC50 dose of Ocoxin in 1% FBS supplemented medium for 48 h as previously described. The selected doses were as follows: PAAD cells with 1:500 of Ocoxin, TNBC with 1:700 of Ocoxin, PRAD with 1:50 of Ocoxin and COAD with 1:700 of Ocoxin. Then, cells were washed with PBS, the total RNA of adhered cells was isolated with the Total RNA purification kit (Norgen, Thorold, ON, Canada) and the quantity and integrity of the RNA was evaluated using the Qubit™ RNA HS Assay Kit (Invitrogen, Waltham, MA, United States) and Agilent RNA 600 NanoChips (Agilent Technologies, Santa Clara, CA, United States).

Afterwards, sequencing libraries were prepared using the TruSeq^®^ Stranded mRNA Library Prep kit, TruSeq^®^ RNA Single Indexes and TruSeq^®^ RNA CD Index Plate (Illumina, San Diego, CA, United States). Then, in order to perform the mRNA sequencing, mRNA was purified, fragmented and primed for cDNA synthesis starting from 1 µg of total RNA with the SuperScriptTM II Reverse Transcriptase (Invitrogen, Waltham, MA, United States) for 10 min at 25°C, 15 min at 42°C, 15 min at 70°C and finished at 4°C. The second cDNA strand was synthesized with Illumina reagents at 16°C for 1 h, then A-tailing and adaptor ligation were performed and enrichment of libraries was achieved by PCR (30 s at 98°C; 15 cycles of 10 s at 98°C; 30 s at 55°C, 20 s at 72°C; 5 min at 72°C and pause at 4°C). Finally, libraries were visualized on an Agilent 2,100 Bioanalyzer using the Agilent High Sensitivity DNA kit (Agilent, Santa Clara, CA, United States) and quantified using QubitTM dsDNA HS DNA kit (Invitrogen, Waltham, MA, United States), quantified using Qubit™ dsDNA HS DNA kit (Invitrogen, Waltham, MA, United States) and sequenced in a NovaSeq 6,000 (Illumina, San Diego, CA, United States).

After, read adapter trimming was performed using fastp (OpenGene, 2022; Chen, 2023; Chen et al., 2018), alignment to reference genome hg38. bwa with STAR (Dobin et al., 2012; Dobin, 2022), PCR duplicates were removed from aligned BAM files with picard (Broad Institute MIT and Harvard, 2022; Dobin et al., 2012), and annotation followed by quantification of reads obtained through mRNAseq was got with SubRead’s FeatureCounts (Liao et al., 2013; Liao et al., 2022). Differential expression analysis was performed with the DESeq2 (Love et al., 2014) version 1.40.2 and the False Discovery Rate correction (FDR) was applied. Heatmaps were done with the library ComplexHeatmap version 2.16.0 (Gu, 2022; Gu et al., 2016).

### 2.4 Analysis of the differentially expressed genes

In order to elucidate whether all the genes altered by Ocoxin were involved in any particular cellular pathway, several analyses were performed by using various softwares based on different databases. First, a systematic analysis of gene functions altered by Ocoxin was performed in each cancer model according to the Kyoto Encyclopedia of Genes and Genomes (KEGG) (Kanehisa and Sato, 2020; Kanehisa and Yoko, 2024). KEGG comprises three databases (PATHWAY, GENES and LIGAND) and enables the analysis of gene functions, linking genomic and functional information. Second, a functional protein association network analysis was performed based on the genes altered by Ocoxin with the STRING software (version 12.0) (Szklarczyk et al., 2023; Snel et al., 2000), which obtains data from Biocarta, BioCyc, GeneOntology, KEGGa and Reactome dabatases in order to predict physical and functional interactions between proteins. Finally, a Venn diagram (Oliveros, 2007) was utilized so as to find whether there were any common altered genes between the four cancer models.

### 2.5 RT-qPCR

Afterwards, the expression level of the genes which were in common in all the four tumors was measured by quantitative reverse transcription polymerase chain reaction (RT-qPCR). Specifically the analyzed genes were: Adrenomedullin 2 (*ADM2*), ChaC glutathione specific gamma-glutamylcyclotransferase 1 (*CHAC1*), Extra spindle pole bodies like 1, separase (*ESPL1*), Heme oxygenase 1 (*HMOX1*), Kinesin family member 20A (*KIF20A*), Metallothionein 1F (*MT1F*), NAD(P)H quinone dehydrogenase 1 (*NQO1*), RAD54 like (*RAD54L*), Sestrin 2 (*SESN2*), Solute carrier family 30 member 1 (*SLC30A1*), Solute carrier family 39 member 10 (*SLC39A10*), Tribbles pseudokinase 3 (*TRIB3*) and Thioredoxin reductase 1 (*TXNRD1*). To do so, cancer cells were treated with Ocoxin for 48 h as previously described and the RNA was again extracted and its quality evaluated as previously described in Section 2.3. Subsequently, 2 μg of mRNA were retrotranscribed into cDNA using the iScript cDNA Synthesis Kit (Bio-Rad, Hercules, CA, United States) and the RT-qPCR was performed using the Itaq Universal SYBR Green Supermix (Bio-Rad, Hercules, CA, United States) on the CFX96 Real-Time System (Bio-Rad, Hercules, CA, United States) and the primers shown on Table 1 (Invitrogen, Waltham, MA, United States). Finally, the relative expression of each gene was standarized to the internal control gene actin beta (*ACTB*).

**TABLE 1:** Primers used for the RT-qPCR.

Gene	Primer	Sequence 5’-3’
*ACTB*	Forward Reverse	CATGTACGTTGCTATCCAGGC CTCCTTAATGTCACGCACGAT
*ADM2*	Forward Reverse	TACACGCAGTGCTGGTACG CTGCTCGTCCAGACATGGC
*CHAC1*	Forward Reverse	GAACCCTGGTTACCTGGGC CGCAGCAAGTATTCAAGGTTGT
*ESPL1*	Forward Reverse	GCTACTTAGTGTCTACCCCACA CAGCAGCATTCCGCAGTAAGA
*GAPDH*	Forward Reverse	ACAACTTTGGTATCGTGGAAGG GCCATCACGCCACAGTTTC
*HMOX1*	Forward Reverse	AAGACTGCGTTCCTGCTCAAC AAAGCCCTACAGCAACTGTCG
*KIF20A*	Forward Reverse	TTGAGGGTTAGGCCCTTGTTA GTCCTTGGGTGCTTGTAGAAC
*MT1F*	Forward Reverse	AATGTAGCAAATGGGTCAAGGTG TCTCCTGCACCTGCGCTGGT
*NQO1 *	Forward Reverse	GAAGAGCACTGATCGTACTGGC GGATACTGAAAGTTCGCAGGG
*RAD54L*	Forward Reverse	TTTACGCCAGAGTCCAGAGTG ATGAAGGCGGAAGGTCTCATA
*SESN2*	Forward Reverse	TCTTACCTGGTAGGCTCCCAC AGCAACTTGTTGATCTCGCTG
*SLC30A1*	Forward Reverse	GGACAACTTAACATGCGTGGA ACACAAAAATCCCCTTCAGAACA
*SLC39A10*	Forward Reverse	ACACCAGATTCTGACTGGCTT GAGGGGATTCTTGTTGGCCT
*TRIB3*	Forward Reverse	TACCTGCAAGGTGTACCCC GGTCCGAGTGAAAAAGGCGTA
*TXNRD1*	Forward Reverse	TAGGACAAGCCCTGCAAGACT CCCCAATTCAAAGAGCCAATGT

## 3 Results

### 3.1 Effect of Ocoxin on the viability of tumor cells

**FIGURE 1:**
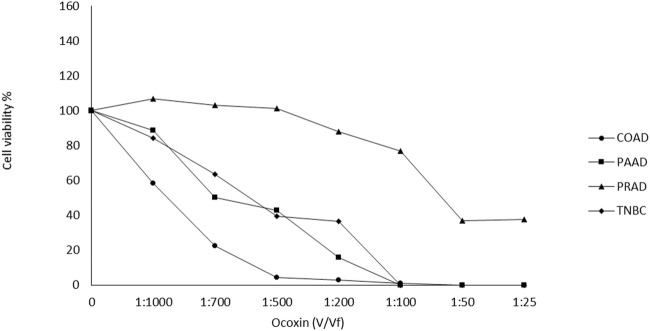
Effect of Ocoxin on the viability of tumor cells. Cancer cells were treated with different dilutions of Ocoxin for 48 h and cell viability was measured with the Cell Counting kit-8.

Ocoxin exhibited a dose-dependent cell viability reduction in all analyzed cell lines after 48 h of treatment. While PRAD cells showed to be quite resistant to Ocoxin up to the 1:50 dilution COAD, PAAD and TNBC were already affected by the natural supplement with the 1:1000 dilution (Figure 1). Approximately, IC50 was reached with the 1:700 dilution in TNBC and COAD cells, with the 1:500 dilution in PAAD cells and with the 1:50 dilution in PRAD cells (Figure 1).

### 3.2 Effect of Ocoxin on the gene expression profile of tumor cells

In order to study whether the effect of Ocoxin on the viability of tumor cells is mediated by genomic alterations, an RNAseq analysis was performed. As shown on Table 2, Ocoxin deregulated the expression of many genes in all the tested cancer cell lines. The number of altered genes was remarkably higher in PAAD cells and in TNBC cells compared to the others. Notably, TNBC exhibited the highest count of deregulated genes after the treatment with Ocoxin, with a total of 3,438 genes altered, followed by PAAD, which showed 1589 altered genes. On the contrary, COAD and PRAD only showed 511 and 594 deregulated genes respectively. Besides that, in COAD, PAAD and TNBC, approximately 50% of the altered genes were upregulated and the other 50% were downregulated, while in PRAD 73% of the deregulated genes were overexpressed.

**TABLE 2:** Number of genes altered by Ocoxin in each cancer type.

COAD		PAAD		PRAD		TNBC	
511		1589		594		3,438	
50% ↓	50% ↑	44% ↓	56% ↑	27% ↓	73% ↑	55% ↓	45% ↑

↓ downregulated; ↑ upregulated.

Afterwards, the differentially expressed gene list was introduced in the KEGG mapper software to determine the KEGG pathways in which all the altered genes are implicated. Later, those pathways were classified according to the percentage of altered genes that belonged to that KEGG pathway versus the total number of deregulated genes in that cancer. As shown on Table 3 the majority of the differentially expressed genes were found to be involved in pathways related to metabolism, cancer and neurodegeneration in all the analyzed cancer types. However, in COAD and PRAD the cell cycle was one of the principal altered pathways, which was not present admist the most deregulated KEGG pathways in PAAD and TNBC.

**TABLE 3:** Main KEGG pathways of each cancer in which all the genes altered by Ocoxin are implicated.

COAD	PRAD	PAAD	TNBC
hsa01100: Metabolic pathways	hsa01100: Metabolic pathways	hsa01100: Metabolic pathways	hsa01100: Metabolic pathways
hsa05200: Pathways in cancer	hsa04110: Cell cycle	hsa05200: Pathways in cancer	hsa05022: Pathways of neurodegeneration
hsa04110: Cell cycle	hsa05200: Pathways in cancer	hsa05022: Pathways of neurodegeneration	hsa05200: Pathways in cancer
hsa05022: Pathways of neurodegeneration	hsa04151: PI3K- Akt signaling pathway	hsa04151: PI3K- Akt signaling pathway	hsa04714: Thermogenesis
hsa05206: MicroRNAs in cancer	hsa05022: Pathways of neurodegeneration	hsa04010: MAPK signaling pathway	hsa05208: Chemical carcinogenesis - reactive oxygen species
hsa04060: Cytokine- cytokine receptor interaction	hsa05208: Chemical carcinogenesis - reactive oxygen species	hsa04060: Cytokine- cytokine receptor interaction	hsa04151: PI3K- Akt signaling pathway
hsa05202: Transcriptional misregulation in cancer	hsa04814: Motor proteins	hsa04810: Regulation of actin cytoskeleton	hsa04010: MAPK signaling pathway
hsa04217: Necroptosis	hsa04510: Focal adhesion	hsa04015: Rap1 signaling pathway	hsa05205: Proteoglycans in cancer
hsa04814: Motor proteins	hsa04218: Cellular senescence	hsa05205: Proteoglycans in cancer	hsa04144: Endocytosis
hsa04210: Apoptosis	hsa04115: p53 signaling pathway	hsa05206: MicroRNAs in cancer	hsa04510: Focal adhesion
hsa03013: Nucleocytoplasmic transport	hsa01240: Biosynthesis of cofactors	hsa04144: Endocytosis	hsa04141: Protein processing in endoplasmic reticulum
hsa04115: p53 signaling pathway	hsa05207: Chemical carcinogenesis - receptor activation	hsa04148: Efferocytosis	hsa04810: Regulation of actin cytoskeleton
hsa03040: Spliceosome	hsa04360: Axon guidance	hsa04510: Focal adhesion	hsa05206: MicroRNAs in cancer
hsa04151: PI3K- Akt signaling pathway	hsa03030: DNA replication	hsa05417: Lipid and atherosclerosis	hsa04015: Rap1 signaling pathway
hsa05207: Chemical carcinogenesis - receptor activation	hsa05202: Transcriptional misregulation in cancer	hsa04714: Thermogenesis	hsa00190: Oxidative phosphorylation
hsa04978: Mineral absorption	hsa04144: Endocytosis	hsa05202: Transcriptional misregulation in cancer	hsa04814: Motor proteins
hsa04510: Focal adhesion	hsa05205: Proteoglycans in cancer	hsa04814: Motor proteins	hsa04218: Cellular senescence
hsa05205: Proteoglycans in cancer	hsa04010: MAPK signaling pathway	hsa05418: Fluid shear stress and atherosclerosis	hsa05417: Lipid and atherosclerosis
hsa04621: NOD- like receptor signaling pathway	hsa04210: Apoptosis	hsa04217: Necroptosis	hsa03010: Ribosome
hsa04657: IL- 17 signaling pathway	hsa04141: Protein processing in endoplasmic reticulum	hsa04150: mTOR signaling pathway	hsa04530: Tight junction

In addition to the aforementioned pathways, PAAD, PRAD and TNBC exhibited differentially expressed genes involved in the MAPK signaling pathway and PI3K-Akt signaling pathways; however, in COAD those pathways were relegated. Moreover, all the 4 cell lines showed alterations in motor proteins, in cancer-related proteoglycans and in genes involved in the focal adhesion and also pathways of miRNAs were found to be deregulated in COAD, PAAD, and TNBC. In turn, many other pathways were, as well, altered in two of the 4 cell lines like for instance the protein processing in endoplasmic reticulum (ER), which was altered in breast and PRAD, and also the p53 signaling pathway and the transcriptional misregulation in cancer in prostate and COAD.

### 3.3 Metabolic genes altered by Ocoxin shared between the cancer models

**FIGURE 2:**
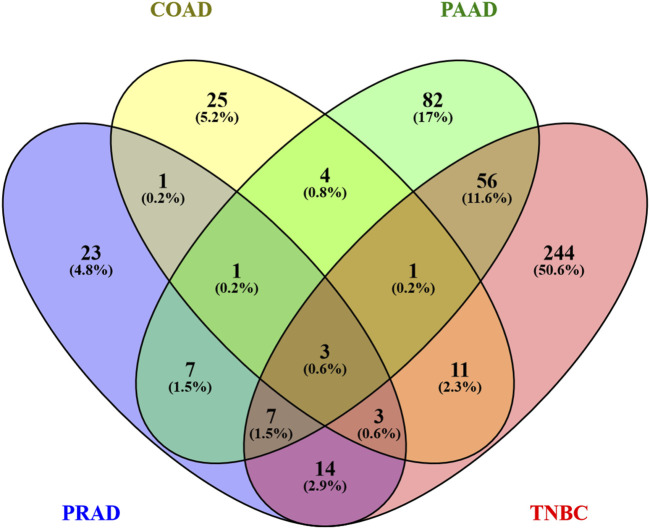
Venn diagram of genes altered by Ocoxin in COAD, PAAD, PRAD, and TNBC that were involved in metabolic pathways. All the genes related to metabolic processes according to the KEGG mapper were included in a Venn diagram in order to compare similarities between the four cancers.

**FIGURE 3:**
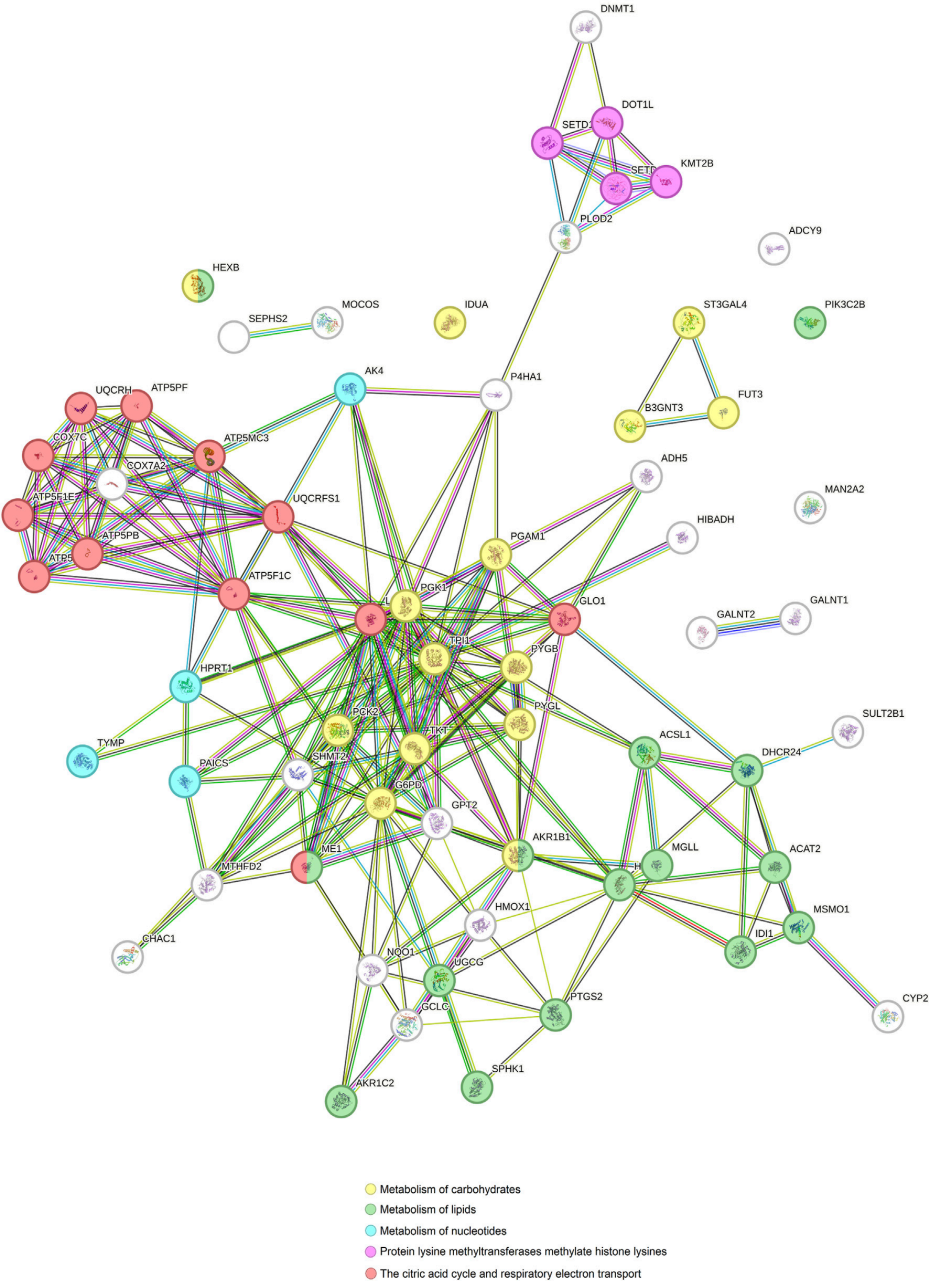
Protein-protein interaction network of genes involved in metabolic processes altered by Ocoxin in common in PAAD and TNBC. All the genes related to metabolic processes altered by Ocoxin in TNBC and PAAD were analyzed through the STRING software in order to analyze the relation between them.

Since the principal pathways altered by Ocoxin were involved in metabolic processes, we compared whether COAD, PAAD, PRAD and TNBC shared the same altered genes related to cell metabolism through a Venn diagram. Figure 2 shows that Ocoxin deregulated three genes in common in all the four cancers: *CHAC1*, *HMOX1* and *NQO1*. Moreover, it was remarkable that PAAD and TNBC shared 67 genes (14%) involved in metabolic pathways, while the rest of paired comparisons shared less than 20 except for PRAD and TNBC, that shared 27 genes (6%). Thus, a more in-depth analysis was performed about those 67 metabolic genes by using the STRING software considering all the genes gathered in the “hsa01100: metabolic pathway” KEGG identifier. The network present in Figure 3 showed that all the genes were linked between them. However, five specific metabolic processes could be identified in the protein-protein interaction network: protein lysine methyltransferases methylate histone lysines, the citric acid cycle and respiratory electron transport and metabolism of nucleotides, lipids and carbohydrates.

### 3.4 Genes altered by Ocoxin shared between the cancer models

**FIGURE 4:**
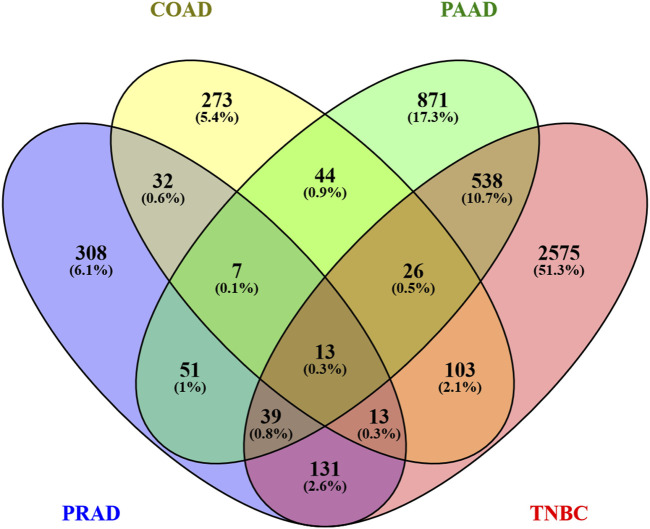
Venn diagram of all the genes altered by Ocoxin in COAD, PAAD, PRAD and TNBC. All the list genes deregulated by Ocoxin were gathered in a Venn diagram.

**FIGURE 5:**
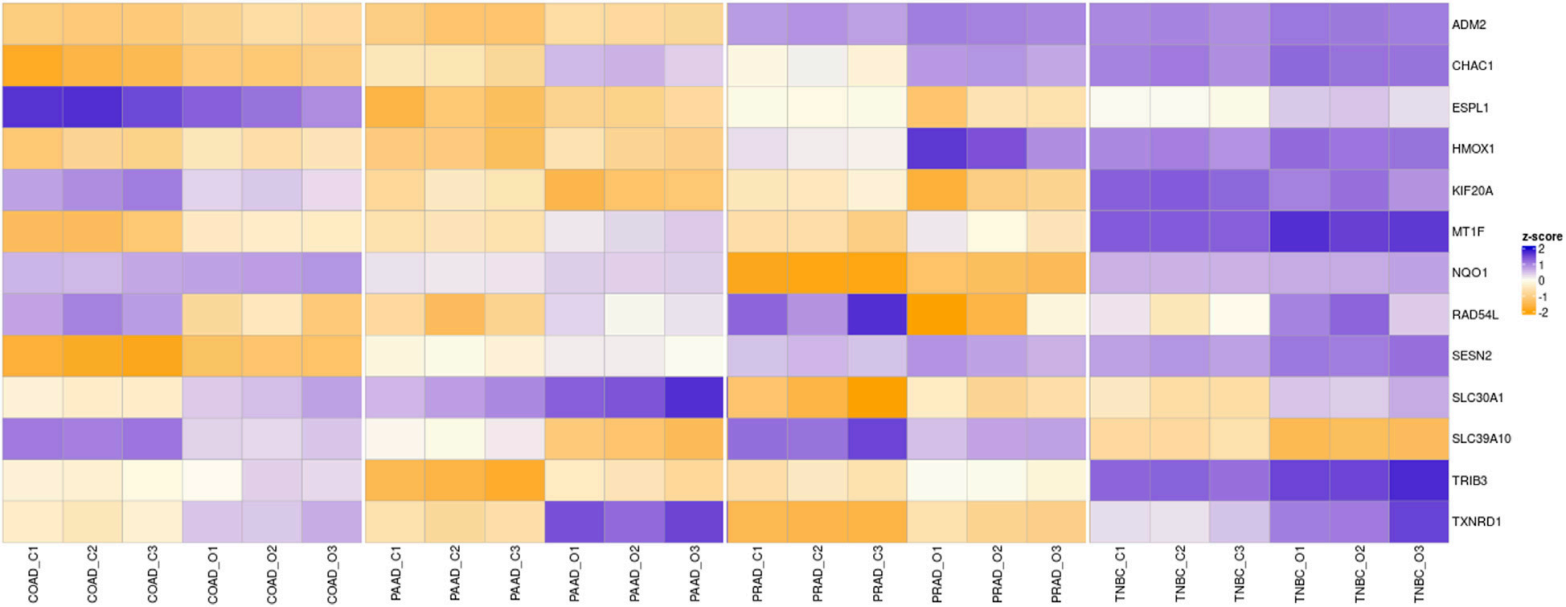
Overview of the RNAseq. The heatmap shows the Z-score of the genes commonly altered by Ocoxin in all the four analyzed cancers. The compound upregulated *ADM2*, *CHAC1*, *HMOX1*, *MTF1*, *NQO1*, *SESN2*, *SLC30A1*, *TRIB3* and *TXNRD1* and downregulated KIF20A and SLC39A10 in all the cancers compared to the untreated samples. *ESPL1* and *RAD54L* were upregulated in PAAD and TNBC and downregulated in PRAD and COAD. Three samples were analyzed for each treatment condition and cancer (C: Control, O: Ocoxin).

Bearing in mind that the four analyzed cancers showed similar altered pathways with the KEGG software, a Venn diagram was utilized in order to determine whether any of the altered genes are shared among the different cancers. As Figure 4 shows, paired comparisons of the genes deregulated by Ocoxin revealed that most of the cancers shared around 5% of the altered genes with each other, while PAAD and TNBC shared 14% of them. Furthermore, 13 genes were found to be altered in common in the four cancers (Figure 4): *ADM2*, *CHAC1*, *ESPL1*, *HMOX1*, *KIF20A*, *MT1F*, *NQO1*, *RAD54L*, *SESN2*, *SLC30A1*, *SLC39A10*, *TRIB3* and *TXNRD1* (Table 4). Moreover, as shown in the heatmap of Figures 5, 11 of those genes were equally deregulated in all the samples, that is, nine genes (*ADM2*, *CHAC1*, *HMOX1*, *MTF1*, *NQO1*, *SESN2*, *SLC30A1*, *TRIB3* and *TXNRD1*) were upregulated and 2 (*KIF20A* and *SLC39A10*) downregulated. On the contrary, two more genes (*ESPL1* and *RAD54L*) showed to be upregulated PRAD and TNBC, and downregulated in COAD and PRAD (Figure 5).

**TABLE 4:** Genes altered in common by Ocoxin in COAD, PAAD, PRAD and TNBC.

Symbol	Name
*ADM2*	Adrenomedullin 2
*CHAC1*	ChaC glutathione specific gamma-glutamylcyclotransferase 1
*ESPL1*	Extra spindle pole bodies like 1, separase
*HMOX1*	Heme oxygenase 1
*KIF20A*	Kinesin family member 20A
*MT1F*	Metallothionein 1F
*NQO1*	NAD(P)H quinone dehydrogenase 1
*RAD54L*	RAD54 like
*SESN2*	Sestrin 2
*SLC30A1*	Solute carrier family 30 member 1
*SLC39A10*	Solute carrier family 39 member 10
*TRIB3*	Tribbles pseudokinase 3
*TXNRD1*	Thioredoxin reductase 1

**FIGURE 6:**
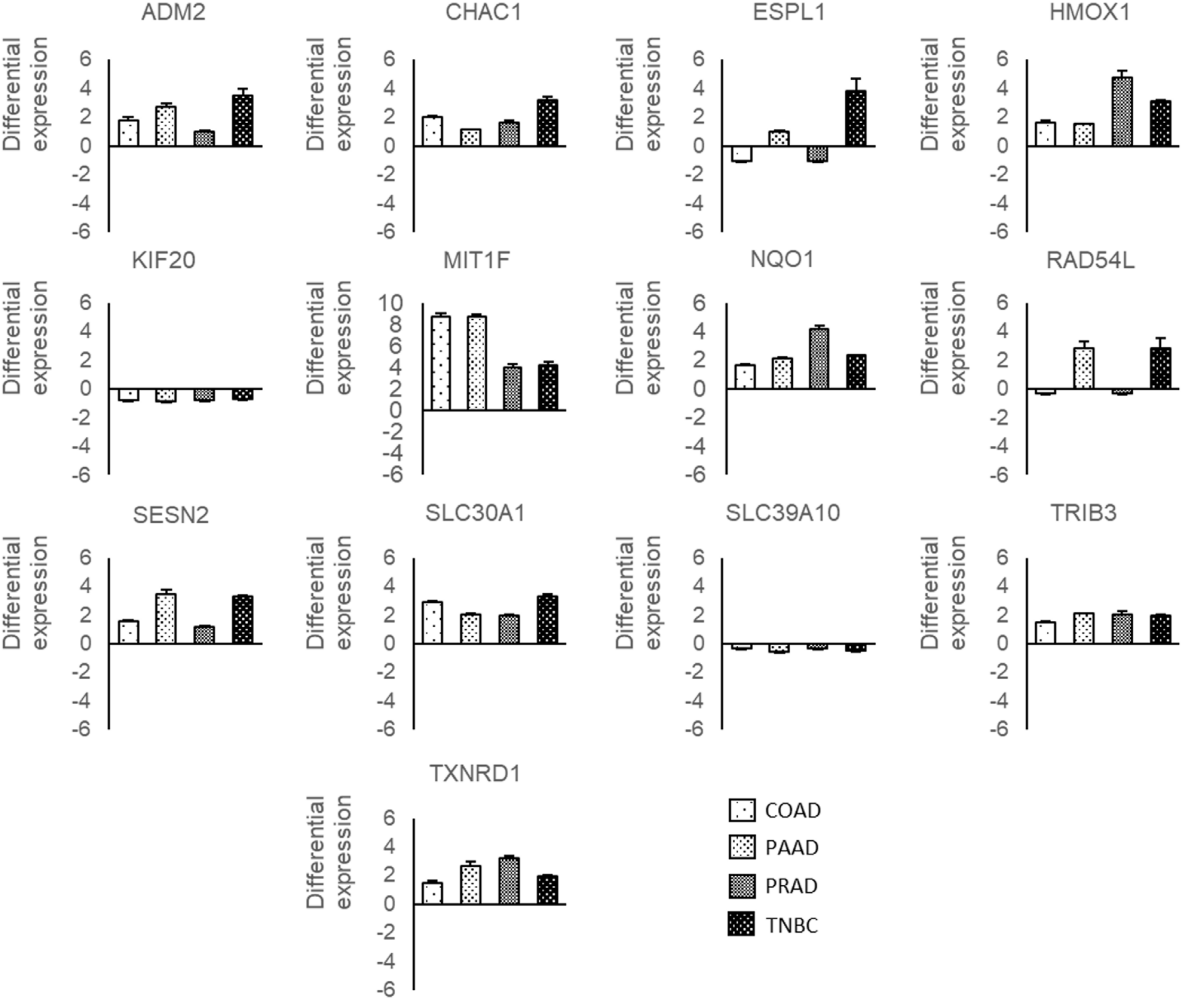
RT-qPCR of genes deregulated by Ocoxin in common in COAD, PAAD, PRAD and TNBC. Cancer cells were treated with the chosen dose of Ocoxin for 48 h (1:700 for COAD, 1:500 for PAAD, 1:50 for PRAD and 1:700 for TNBC), total mRNA was extracted and a RT-qPCR was carried out. Data were normalized to the actin beta expression of each tumor.

Later, the expression level of those 13 genes deregulated by Ocoxin in the four cancers was validated through RT-qPCR. As shown in Figure 6, *ADM2*, *CHAC1*, *HMOX1*, *MT1F*, *NQO1*, *SESN2*, *SLC30A1*, *TRIB3,* and *TXNRD1* were all upregulated in all the cancers while KIF20A and SLC39A10 were downregulated. Conversely, *ESPL1* and *RAD54L* showed a different alteration pattern among the tumors being both upregulated in PAAD and TNBC and downregulated in COAD and PRAD.

**FIGURE 7:**
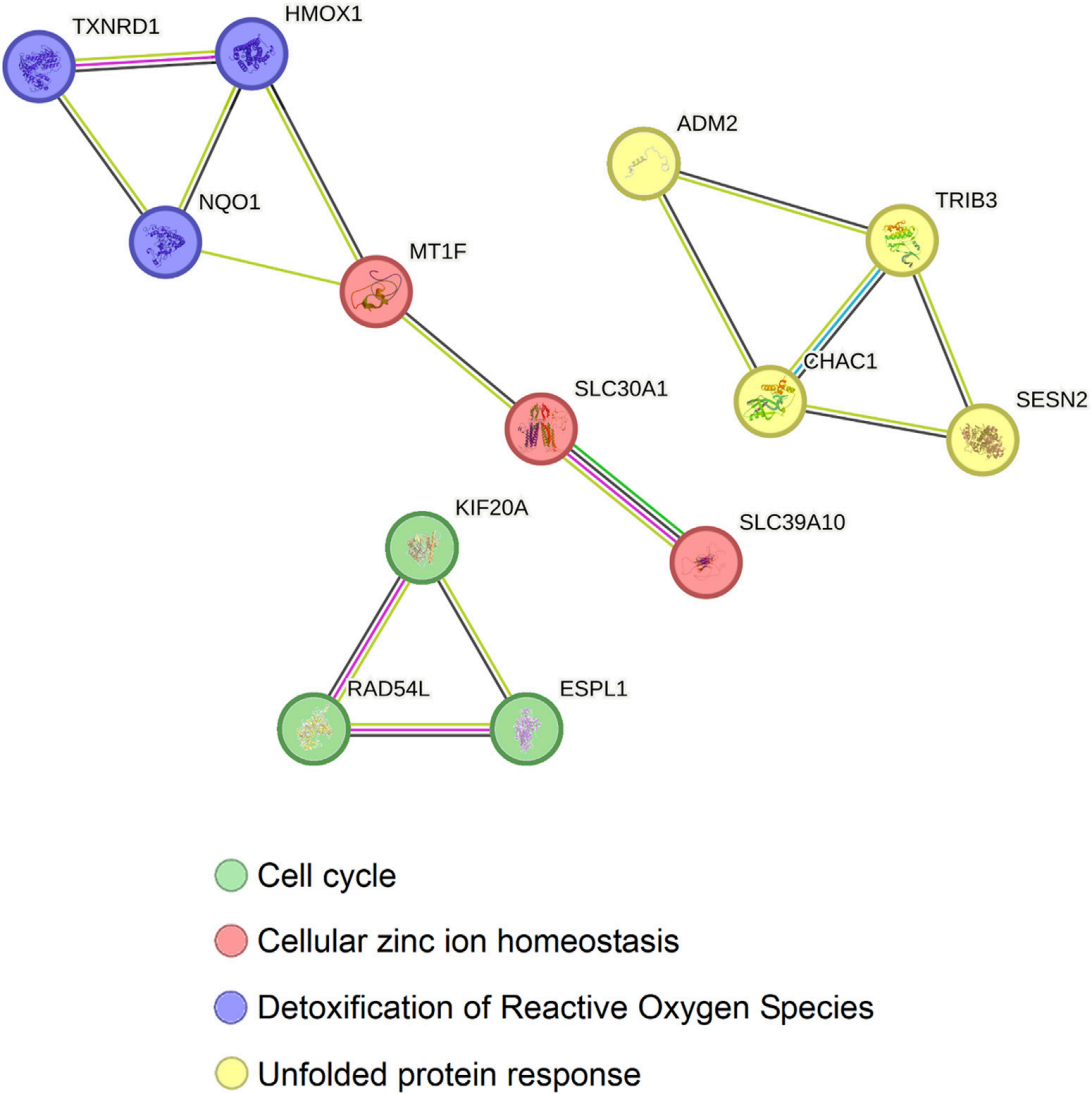
Protein-protein interaction between the 13 genes commonly altered by Ocoxin in COAD, PAAD, PRAD, and TNBC. All the genes altered by Ocoxin in common in the four cancers were analyzed through the STRING software in order to analyze the relation between them. Gene interactions were classified in three independent groups with four biological functions.

Afterwards, to further understand what the common genes altered by Ocoxin in TNBC, PAAD, PRAD and COAD are involved in, a protein-protein interaction analysis was performed through STRING. Figure 7 shows that the 13 proteins can be classified in three independent groups which are related to four protein biological functions. The first group is comprised of the proteins, TXNRD1, HMOX1 and NQO1, which are involved in the detoxification of Reactive Oxygen Species (ROS) and are related to MT1F, SLC30A1 and SCL39A10, proteins involved in Zinc homeostasis. The second group, constituted by ADM2, TRIB3, CHAC1 and SESN2, is involved in the unfolded protein response (UPR) and finally the third group contains KIF20A, RAD54L and ESPL1, which are involved in the cell cycle.

**FIGURE 8:**
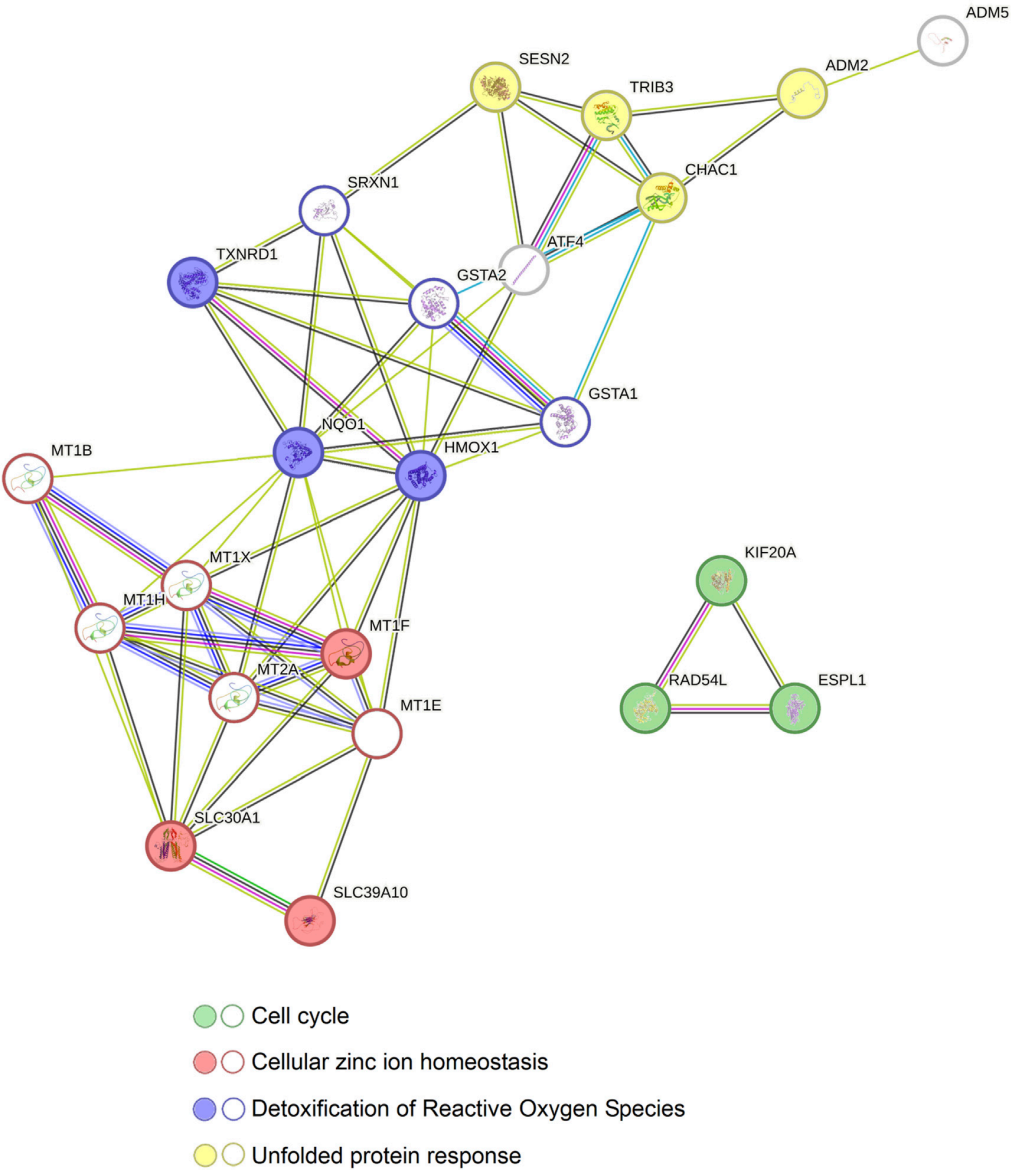
Relation between the protein-protein interaction networks of the genes altered by Ocoxin in common in COAD, PAAD, PRAD, and TNBC. The STRING software was used to reveal the connection between all the genes altered by Ocoxin in common in all the cancers. The 13 genes altered by Ocoxin in common in all the tumors (circles with colored background) were interconnected through nine new nodes (circles without a colored background) and classified in four biological functions.

Afterwards, in order to examine the potential correlation between those proteins, we increased the number of predicted nodes and observed that all the proteins show to be correlated except for ESPL1, KIF20A and RAD54L through nine new nodes (Figure 8). As in the previous results, the proteins related to zinc homeostasis (MT1F, SLC30A1 and SLC39A10) together with more metallothioneins (MT1B, MT1E, MT1H, MT1X and MT2A) showed to be interconnected with the proteins involved in detoxification of ROS and, likewise, those molecules were linked to the proteins of the genes that take part in the UPR (ADM2, CHAC1, TRIB3 and SESN2) via the activating transcription factor 4 (ATF4), the glutation-S-transferases A1 and A2 (GSTA1 and GSTA2) and the sulfiredoxin 1 (SRXN1) (Figure 8).

## 4 Discusion

Cancer is one of the most worrisome pathologies in the world. Nowadays, due to the prevention schedules, advances in oncological surgery and the identification of new therapeutic alternatives have permitted to treat, prolong and improve the life quality of patients suffering from many cancers. However, despite the progress, many of them experience recurrence and metastasis and although they can be treated with radiotherapy or chemotherapy, the adverse effects arising from those treatments considerably damage their quality of life. Thus, the latest research is focused on the identification of new therapeutic alternatives that not only treat cancer but also extend life expectancy and improve the wellbeing of cancer patients by reducing the undesired effects caused by the treatments. In this scenario, there is growing interest in the application of bioactive compounds of natural origin, such as, EGCG, curcumin, resveratrol, quercetin and many others, to treat different kinds of cancers and to reduce the non-desirable side effects produced by chemotherapy (Naujokat and McKee, 2021; Ding et al., 2020; Rana et al., 2021; Vladu et al., 2022; Maleki Dana et al., 2022).

Ocoxin is a natural mixture composed of plant extracts, amino acids, vitamins and minerals known for its established antioxidant, anti-inflammatory and immunoregulatory properties, which has further demonstrated to exert antitumor effects in several cancer models *in vitro* and *in vivo* by impairing tumor development at different levels. On the one hand, Ocoxin has reduced cell viability *in vitro* by decreasing cell proliferation and inducing apoptosis and cell cycle arrest in many cancers. On the other hand, the compound has increased the cytotoxic effect of first-line chemotherapeutic drugs while reverting the resistance to chemotherapy caused by CAFs (Hernandez-Unzueta et al., 2017; Benedicto et al., 2021; Hernandez-Unzueta et al., 2019). Besides, Ocoxin has been used in clinics and showed an improvement in the quality of life of patients with hepatocellular carcinoma, lung cancer, PRAD, PAAD, cervical cancer and endometrial adenocarcinoma, among others, while increasing survival in some patients (Al-Mahtab et al., 2015; Kaidarova et al., 2019; Ruiz Lorente et al., 2020; Lima-Pérez et al., 2024; García-Perdomo et al., 2021). Therefore, in the present work, we aimed to deepen into the fundamental mechanisms through which Ocoxin exerts its antitumoral activity against cancer, as well as to identify whether there are any commonalities among the various types of cancer that may offer insights into the functionality of the nutritional supplement.

In relation to that, previous works have described the role of natural products acting as multi-targeted elements to alter different signaling pathways by regulating gene expression or targeting proteins and their secretion (Luesch and Paavilainen, 2020; Irshad and Husain, 2021; Nasir et al., 2020). Considering that cancer is a dynamic disease and that tumor cells possess distinct molecular signatures and sensitivity to therapies, the capacity of bioactive compounds to target cancer through several mechanisms at once needs to be explored since those effects might be an interesting approach to personalized therapies. Therefore, once tumor and patient’s characteristics are defined, instead of using established first-line treatments, those could be combined with natural products, thereby increasing the potential for a successful treatment.

According to our results, the mixture altered several genes in each type of cancer. Notably, TNBC and PAAC showed a substantially higher number of significantly altered genes, 3,438 and 1589 respectively, from which 12% were in common. Contrarily, in PRAD and COAD the number of deregulated genes was around 500 in each cancer. Besides, taking into consideration all the results, Ocoxin deregulated the expression of 13 genes in common in the analyzed tumors, which can be classified in three principal independent groups based on four functional protein association networks. The first group is composed of proteins related to the UPR (ADM2, CHAC1, SESN2, TRIB3). The second group gathers proteins involved in two biological processes such as, zinc homeostasis (MT1F, SLC30A1, SLC39A10) and detoxification of ROS (HMOX1, NQO1, TXNRD1). These molecules can also be englobed as part of the nuclear factor erythroid 2-related factor 2 (NRF2) pathway, which is responsible for the defense against oxidative stress and the maintenance of stable conditions. Finally, the third group contains proteins related to the cell cycle (ESPL1, KIF20, RAD54L). In this context, the upregulation of the genes encoding for the proteins of the first and second group that deal with cell homeostasis could have led to the production of misfolded proteins that are not correctly eliminated by the ER-associated degradation (ERAD) (Smith et al., 2011; Travers et al., 2000), causing ER stress. In fact, some natural bioactive compounds are known to induce or reduce ER stress. Actually, Xue et al. reported that morusin, a flavonoid obtained from white mulberry, induces ER stress on human epithelial ovarian cancer cells by upregulating the heat shock protein family A member 5 (*HSPA5*), the DNA damage inducible transcript 3 (*DDIT3* or *CHOP*), the endoplasmic reticulum to nucleus signaling 1 (*ERN1*) and the α subunit of the translation initiation factor 2 (*eIF2α*) driving cells to a paraptosis-like cell death (Xue et al., 2018) and Zhang et al. observed that curcumin boosts ER stress-associated apoptosis on human papillary thyroid carcinoma cells (Zhang, L. et al., 2018).

It is known that cells, in order to restore the proteostasis, activate a mechanism named the UPR, whereby modulation of the gene expression occurs and protein synthesis becomes diminished (Harding et al., 2000; Ron and Walter, 2007). In this scenario, cells need to make a decision: to continue living or to die. This choice is controlled by *eIF2α*, whose activation reduces the global protein synthesis and, at the same time, induces the activating transcription factor 4 (*ATF4*) (Harding et al., 2003). These genes are central players of the integrated stress response (ISR), an adaptive response by which cells have the ability to reprogram gene expression in order to provide an optimized response to deal with stressful conditions. Despite the fact that the ISR is mainly a homeostatic pro-survival mechanism, cell response will depend on the nature and intensity of the driving stimuli, which, in case of prolonged and severe signals, will drive cells to death (Pakos-Zebrucka et al., 2016). In fact, this signaling network could be useful to face cancer as it is known to contribute to drug sensitivity and to promote the expression of immunotherapeutic targets (Obiedat et al., 2020; Lines et al., 2023; Tian et al., 2021). Actually, over the last years ISR modulating therapies have attracted significant attention to such an extent that diverse ongoing clinical trials target different levels of this pathway (Lines et al., 2023). During the ISR, various kinases sense different stimuli, such as nutrient deprivation, heme deficiency, DNA damage, oxidative stress or ER stress and converge on the phosphorylation of eIF2α leading to the induction of the *ATF4* (Lines et al., 2023; Tian et al., 2021). In this work, the treatment with Ocoxin only upregulated *ATF4* in PAAD. However, *ATF3*, *ATF5, PMAIP1*, *DDIT4* and *CHOP*, which are downstream genes of *ATF4*, were upregulated by Ocoxin in at least one of the studied cancers, while *TRIB3* was overexpressed in all of them. Furthermore, despite the fact that *ATF4* itself was not deregulated by Ocoxin in all the analyzed cancer models, it arises as a central protein in the correlation among the genes altered by the compound (except for *KIF20A*, *RAD54L* and *ESPL1*) in common in COAD, PAAD, PRAD and TNBC. In fact, most of the genes upregulated by Ocoxin, such as *ADM2*, *CHAC1*, *HMOX1*, *MTF1*, *NQO1*, *SESN2*, *TRIB3* or *TXNRD1*, are related to the ER stress either directly or indirectly. On the one hand, *ADM2*, *CHAC1*, *SESN2* and *TRIB3* are stress sensors of UPR controlled by the *ATF4* gene under ISR (Crawford et al., 2015; Kovaleva et al., 2016; Garaeva et al., 2016; Kalinin et al., 2023) and on the other hand, *HMOX*, *MTF1*, *NQO1* and *TXNRD1* are involved in ROS detoxification, whose imbalance causes ER stress (Shi et al., 2023; Dunn et al., 2014; Li, H. et al., 2021; Rodríguez-Menéndez et al., 2018; Scuto et al., 2022). Furthermore, *MTF1* encodes for a zinc dependent transcription factor that responds to changes produced in the intracellular heavy-metal homeostasis. Briefly, *MTF1* regulates cellular processes by binding zinc to metallothioneins (MTs) or through the transcription of metal transporters of the ZRT/IRT-like protein (ZIP) family and the zinc transporter (ZnT) family (Eide, 2006; Lichten et al., 2011; Lichten and Cousins, 2009). While ZIP family carriers import zinc from the extracellular space or subcellular organelles to the cytoplasm, ZnT family components export the metal from the cytoplasm to the extracellular milieu or organelles (Sun, C. et al., 2024; Cotrim et al., 2019). At this point, it needs to be mentioned that Ocoxin contains high levels of zinc, which could have altered the expression of genes that participate in its homeostasis. In particular, those genes deregulated by Ocoxin were *SLC30A1* and *SLC39A10* (also known as *ZNT1* and *ZIP10*), which enable zinc discharge and acquisition respectively. In this regard, Ocoxin upregulated the expression of *SLC30A1*, whereas downregulated that of *SLC39A10*, resulting in an increase of export to the extracellular compartment and a reduction of intracellular zinc accumulation (Fukada and Kambe, 2011; Palmiter and Findley, 1995). Therefore, cancer cells may activate *MTF1* in the presence of high concentrations of this metal, causing an underexpression of *SLC39A10* and an overexpression of *SLC30A1* in order to recover zinc homeostasis. In fact, this metal is an essential component for the correct functioning of the ER since any homeostatic disruption can originate ER stress and subsequent UPR activation, which, as previously described, are related to the ISR pathway (Homma et al., 2013; Nguyen et al., 2013; Kambe et al., 2017). Therefore, we deem that Ocoxin drives tumor cells to a stressful environment through the ISR. Bearing in mind the intertumoral and intratumoral heterogeneity, and given the implication of the ISR in cellular adaptation and tumor development, modulating its activity could be a formidable treatment for many cancers, especially in combination with other therapies. Due to the multiple cellular mechanisms controlled by the ISR, the manipulation of this pathway could reach several populations of cells with distinct genetic signatures within the same tumor, enabling the overcoming of barriers such as treatment resistance or relapse, which represents a major impediment in medical oncology.

Anyway, it is crucial to consider that our results correspond to cells that had been treated with Ocoxin for 48 h, a period that might not be sufficient for the cells to determine their fate. Probably, as described before, if the stress caused by Ocoxin persisted, cells would engage cell death signaling pathways. This is supported by unpublished data where we observed that the mixture led to an increase in the percentage of dead cells after 72 h, thereby confirming that cells ultimately succumb to death after sustained stress conditions. Nevertheless, all the analyzed tumors exhibited either unchanged or downregulated caspases, indicating that cells may be undergoing a caspase-independent form of programmed cell death or that a duration exceeding 48 h is required for the activation of apoptotic caspases. Here, we show that, among other processes, Ocoxin deregulated the expression of multiple genes associated with autophagy, necroptosis and especially ferroptosis, a type of programmed cell death characterized by the accumulation of free iron within cells and by lipid peroxidation. Due to its clinical importance, ferroptosis has gained attention over the last years as an apoptosis-independent programmed cell death. This mechanism is strongly associated with metabolic processes, such as, carbohydrate metabolism, lipid metabolism and amino-acid metabolism (Ye et al., 2024) as well as iron and selenium metabolism (Conrad and Proneth, 2020) among others. In this regard, keeping in mind that cell metabolism is a crucial process to sustain cell growth and self-renewal, it leads to the fact that metabolic activities and the metabolites produced in the tumor could have an influence on ferroptosis (Jiang et al., 2024). Interestingly, Ocoxin caused an overexpression of *HMOX1*, a critical mediator in ferroptosis that promotes cell death in the presence of oxidative stress and high iron concentrations inside the cells (Chiang et al., 2018). Moreover, the nutritional supplement also deregulated the expression of *NQO1*, a gene responsible for redox control that is also involved in ferroptosis (Zhang, L. et al., 2018; Wang W. et al., 2023). Actually, both *HMOX*1 and *NQO1* are downstream genes of *NRF2*, which play a central role in the NRF2 pathway defending cells against ROS. In consonance, Liu et al. (2022) described that Vitamin C, which is one of the components of Ocoxin, sensitized pancreatic cancer cells to erastin-induced ferroptosis by activating the AMPK/NRF2/HMOX1 pathway (Liu, Y. et al., 2022). In line with this, Wang et al. reported that eriodictyol, a flavonoid obtained from certain plants, regulated ferroptosis through the NRF2/HMOX1/NQO1 signaling pathway in ovarian cancer (Wang X. et al., 2023). Thus, the overexpression of *HMOX1* and *NQO1* observed in cancer cells treated with Ocoxin could have accounted for the subsequent mortality of those cells when exposed to the compound. Besides, it is important to bear in mind that *HMOX1* and *NQO1* are also two of the genes related to the main pathway deregulated by Ocoxin in all the four analyzed cancers: cell metabolism. Interestingly, both *HMOX1* and *NQO1* are downstream genes of *ATF4* and, therefore, they are implicated in the ISR pathway mentioned before. Wang W. et al. (2023) reported that a flavonoid derived from licorice increased HMOX1 and decreased metabolic glutathione peroxidase 4 (GPX4), which reduces lipid peroxidation, leading to ferroptosis (Wang X. et al., 2023; Koppula et al., 2021; Zhao et al., 2019; Li, S. et al., 2010). Additionally, Yang and colleagues (2021) described that the suppression of the KIF20A/NUAK1/NRF2/GPX4 signaling pathway induces ferroptosis and increases the sensitivity of COAD to chemotherapy. Interestingly, although the expression of *NUAK1*, *NRF2* and *GPX4* was not altered by Ocoxin, the supplement downregulated the expression of *KIF20A* in the four tumors (Yang et al., 2021). Nevertheless, a deregulation of *SLC7A11* was detected in PRAD, PAAD and COAD, which is a downstream gene of NRF2 and the ISR (Koppula et al., 2021). This gene encodes for a cysteine/glutamate antitransporter system composed of two subunits (*SLC7A11* and *SLC3A2*) that exchange extracellular cysteine for intracellular glutamate and regulate ferroptosis through the glutathione/glutathione peroxidase 4 (GSH/GPX4) pathway. Here we observed that Ocoxin upregulated *SLC7A11* in three cancers along with *SLC3A2* in PAAD, prompting an accumulation of cysteine, which is a component of Ocoxin, and a loss of glutamate inside the cell, impeding the function of GPX4 and, therefore, contributing to ferroptosis (Koppula et al., 2021). Correspondingly, Tang et al. described how certain metabolites obtained from plants used in Chinese medicine altered *SLC7A11* and *GPX4*, among others, which reduced cancer development (Tang et al., 2024). Moreover, Ocoxin overexpressed *CHAC1*, a gene that also participates in the glutathione degrading metabolism, which curiously was connected to HMOX1 and NQO1 through ATF4 in the protein-protein interaction network analysis. According to several authors, CHAC1 is essential for the maintenance of NRF2 activation and is also involved in ferroptosis (Kreß et al., 2023; Kreß et al., 2023). Actually, He et al. reported that CHAC1 plays a role in hindering tumor development and enhancing the efficacy of docetaxel in prostate cancer by triggering ferroptosis and ER stress (He et al., 2021) and additionally, Chen et al. reported that this gene facilitates ferroptosis as well as necroptosis in breast cancer (Chen, M. et al., 2017). Furthermore, this is corroborated by Liu et al., who described the role of CHAC1 inducing ferroptosis through the glutathione metabolism in retinal pigment epithelial cells (Liu et al., 2023). Taking that into consideration, Ocoxin could have decreased intracellular glutathione concentration through the overexpression of CHAC1 and the subsequent glutathione degradation, driving cells to ferroptosis. Thus, we conclude that Ocoxin induces ferroptosis in tumor cells through several pathways, which could be beneficial for patients that do not respond to chemotherapy. According to several authors, apoptosis-resistant cancer cells have showed to be susceptible to ferroptosis inducers (Hangauer et al., 2017; Tsoi et al., 2018; Viswanathan et al., 2017). In this manner, a greater efficacy of current therapies could be achieved leading to the induction of apoptosis while simultaneously targeting resistant cells via ferroptosis. Moreover, this type of cell death has also been associated with the tumor microenvironment by several authors. On the one hand, Costa da Silva et al. reported that iron uptake by M2-like tumor-associated macrophages drives a differentiation to M1-proinflammatory phenotype which helps to reduce tumor growth (Costa da Silva et al., 2017). On the other hand, Wang et al. described that immunotherapy-activated CD8^+^ T cells enhance tumor ferroptosis by inhibiting the expression of molecules implicated in the glutamate–cystine antiporter system through interferon gamma (IFNγ) release, which causes lipid peroxidation and leads tumor to ferroptosis (Wang et al., 2019). In this regard, we previously mentioned that Ocoxin has an antitumor effect in different cells of the tumor stroma. However, additional analyses need to be carried out so as to identify the possible implication of the compound in the induction of ferroptosis through the tumor microenvironment. Nonetheless, the use of Ocoxin as an adjuvant agent could enhance first-line therapies by targeting the different populations of cancer cells and the tumor microenvironment, thereby increasing the susceptibility of therapy-persistent cancer cells to common therapies and preventing tumor recurrence and metastasis.

Nevertheless, those are not the unique metabolic genes targeted by Ocoxin. As previously mentioned, TNBC and PAAC shared a considerable number of altered genes. In particular, considering only those genes involved in metabolic pathways, those two cancers showed 14% of altered genes in common, while at the same time those were not altered in PRAD and COAD. In this regard, it is important to bear in mind that the analyzed PAAD and TNBC cells come from primary tumors, while those of PRAD and COAD were isolated from metastatic sites. A deeper analysis about the specific metabolic pathways where the genes altered by Ocoxin are involved, showed that in the primary tumors (PAAD and TNBC) the compound targeted genes from the carbohydrate, nucleotide and lipid metabolism, protein lysine methyltransferases (PKMTs) methylate histone lysine and the citric acid (TCA) cycle and respiratory electron transport. Specifically, regarding the respiratory electron transport, Ocoxin downregulated the expression of the mitochondrial oxidative phosphorylation (OXPHOS) genes *ATP5F1C*, *ATP5MC3*, *ATP5F1E*, *ATP5PB*, *ATP5PF* and *ATP5MG*, which could have been also driven by the ISR. Briefly, the phosphorylation of *eIF2α* mediated by the ISR stimulates the transcription of *CHOP*, *ATF4* and *ATF5* genes that participate in the activation of the mitochondrial UPR (UPR^mt^) (Zhang, G. et al., 2021; Ogilvie et al., 2005; Boyce et al., 2005) Since both, the ISR and the UPR^mt^, are two strongly related processes that function together to maintain the mitochondrial metabolism and function, we can assume that Ocoxin altered mitochondrial homeostasis. Interestingly, central players of this pathway, *SIRT7* and *HSP60* (*HSPD1*) (Lu et al., 2022), are overexpressed in the primary PAAD and TNBC but not in the metastatic COAD and PRAD. A similar phenomenon was also observed in this study, while the KEGG enrichment analysis indicated that cell cycle is one of the principal pathways altered in the metastatic PRAD and COAD tumors, in PAAD and TNBC the number of genes involved in the cell cycle was not that remarkable. In this context, it is widely described that one of the antitumor mechanisms of Ocoxin is the alteration of the cell cycle (Díaz-Rodríguez et al., 2018; Diaz-Rodriguez et al., 2017; Diaz-Rodriguez et al., 2016; Benedicto et al., 2021; Hernandez-Unzueta et al., 2023; Hernandez-Unzueta et al., 2019; Hernández-García et al., 2015; Pérez-Peña et al., 2019; Márquez et al., 2016). Among the 13 genes commonly altered by the mixture in the four cancer types, three of them, *KIF20A*, *ESPL1* and *RAD54L* are implicated in the cell cycle progression. Curiously, although *KIF20A* is downregulated in all tumors, *ESPL1* and *RAD54L* are differentially expressed between primary and metastatic tumors, being upregulated in PAAD and TNBC while downregulated in COAD and PRAD. We previously discussed the relation of *KIF20A* with ferroptosis; however, this gene mediates the spindle formation during mitosis and its overexpression is related to both, cancer development and a poor overall survival of patients. In fact, *KIF20A* is overexpressed in all the cancers present in the Gene Expression Profile Interactive Analysis (GEPIA) server (Tang et al., 2017), including COAD, PAAD, PRAD, and TNBC. Interestingly, in this work we demonstrated that Ocoxin downregulated *KIF20A* in all the analyzed tumors, that is, the compound reverted the expression of this gene. Besides, previous works report that the nutritional mixture also slowed down the cell cycle of tumor cells by causing an arrest on the G2/M phase in breast cancer while in colorectal carcinoma also affected the cell transition from G1 to S phase (Hernández-García et al., 2015; Márquez et al., 2016). Moreover, it is known that the inhibition of *KIF20A* hinders the growth of several cancer cells (Jin et al., 2023) and Nakamura and colleagues reported that the suppression of *KIF20A* arrested cells in the G2/M phase in breast cancer, while Zhang et al. reduced the colorectal cancer cell proliferation and migration by drecreasing the cell transition from G1 to S phase through the *E2F1* transcription factor in hepatocellular carcinoma (Nakamura et al., 2020; Wang, H. et al., 2020). Considering all this, the reduction of *KIF20A* expression could be a specific therapeutic strategy to treat cancer, which proves that the understanding of factors such as the genetic profile of the patient and the mechanism of action of the compound might be a step towards more personalized therapies. In this regard, several authors have reported so far that Ocoxin slows down the cell cycle in different phases depending on the tumor type. Taking this into consideration, we can suggest that *ESPL1* and *RAD54L*, which are differentially expressed between the primary TNBC and PAAC and the metastatic PRAD and COAD, are modulating the cell cycle in various ways. While *RAD54L* belongs to the DEAD-like helicase superfamily and is involved in the homologous recombination and DNA repair, *ESPL1*, also known as separase, initiates the final separation of sister chromatids during anaphase. *ESPL1*, which is typically upregulated in tumor tissue due to the rapid proliferation of cancer cells, is controlled by the *PTTG1* regulator of sister chromatid separation, also known as securin, which has the ability to inhibit *ESPL1* (Sun, Y. et al., 2009; Zhong et al., 2023). Here we observed that, on the one hand, the treatment with Ocoxin upregulated *ESPL1* and downregulated *PTTG1* in TNBC and PAAD, and on the other hand, *ESPL1* was downregulated in PRAD and COAD, but *PTTG1* was not altered. Moreover, while most of the cell cycle genes in common in metastatic tumors, such as, *MCM2*, *MCM3*, *MCM4*, *MCM5*, *MCM7*, *BUB1*, *BUB1B* and *AURKB* are downregulated, including *CDKN2B*, which is downregulated in TNBC and *CDKN2C* in PAAC, both encoded proteins that function as a cell growth regulator that controls cell cycle G1 progression. Nevertheless, most of the cell cycle genes of the metastatic COAD and PRAD tumors were upregulated, including the abovementioned *CDKN2B*, showing that Ocoxin regulates the G1 phase of the cell cycle through different routes.

To conclude, we can suggest that Ocoxin shows different mechanisms of action depending on the type and stage of the tumor. In fact, the compound demonstrated a greater sensitivity to primary tumors, where it altered more genes than in metastatic tumors. Nevertheless, the main altered pathway was cell metabolism, with a significant emphasis on the process of ferroptosis among the genes modulated by Ocoxin in common in all the studied cancers. Moreover, it must be mentioned that Ocoxin exerted a varied effect in each cancer by acting on distinct pathways, which, considering tumor heterogeneity, might reflect the multi-target action of the compound. Although the primary process modulated by the supplement was cell metabolism, the effect of Ocoxin in metastatic tumors exerted a substantial effect on the cell cycle. Therefore, we can presume that the antitumor action of Ocoxin is, in part, attributable to mechanisms englobed in the UPR, ISR and metabolic alterations leading to cell death, which may also explain its supportive role in chemotherapy. Modulating those pathways could be a crucial strategy to provide more specific treatments in clinical settings and improve oncological therapies, especially in aggressive or treatment-resistant tumors. However, further studies are needed to delve into the antitumoral mechanism of Ocoxin.

## Data Availability

The data presented in the study are deposited in the GEO repository, accession number GSE291188.
